# Protective Effect of Enalapril against Methionine-Enriched Diet-Induced Hypertension: Role of Endoplasmic Reticulum and Oxidative Stress

**DOI:** 10.1155/2015/724876

**Published:** 2015-11-12

**Authors:** Yanfen Zhou, Lianyou Zhao, Zhimin Zhang, Xuanhao Lu

**Affiliations:** ^1^Department of Cardiology, Tangdu Hospital, Fourth Military Medical University, Xi'an 710038, China; ^2^Intervention Therapy Department of Vascular Disease, Chinese Medicine Hospital of Shaanxi Province, Xi'an 710003, China

## Abstract

In the present study, we investigated the effect of methionine-enriched diet (MED) on blood pressure in rats and examined the protective effect of enalapril, a widely used angiotensin converting enzyme inhibitors (ACEi) class antihypertensive drug. The results showed that MED induced significant increase of SBP and Ang II-induced contractile response in aortae of rats. MED significantly increased plasma levels of homocysteine (Hcy) and ACE. In addition, MED increased the phosphorylation of protein kinase R-like endoplasmic reticulum kinase (PERK) and eukaryotic initiation factor 2 (eIF2*α*) and expression of activating transcription factor 3 (ATF3) and ATF6 in aortae of rats, indicating the occurrence of endoplasmic reticulum (ER) stress. Moreover, MED resulted in oxidative stress as evidenced by significant increase of TBARS level and decrease of superoxide dismutase and catalase activities. Administration of enalapril could effectively inhibit these pathological changes induced by MED in rats. These results demonstrated that ACE-mediated ER stress and oxidative stress played an important role in high Hcy-induced hypertension and MED may exert a positive loop between the activation of ACE and accumulation of Hcy, aggravating the pathological condition of hypertension. The data provide novel insights into the mechanism of high Hcy-associated hypertension and the therapeutic efficiency of enalapril.

## 1. Introduction

Hypertension is viewed globally as a chronic pathological condition characterized by systemic and persistent elevation of arterial pressure. It is believed that prolonged, uncontrolled hypertension leads to cardiovascular diseases and various organ damage cases. Hypertension-related health problems contribute to 13% of global deaths [1]. Increased blood homocysteine (Hcy) level is believed to be an independent risk factor for coronary heart disease, stroke, and peripheral vascular disease [2]. Experimental and clinical evidence has shown that hyperhomocysteinemia is positively associated with hypertension [3].

Cysteine, Hcy, and methionine belong to a group of sulfur-containing amino acids. Methionine functions as a precursor of S-adenosyl methionine (SAM) and a main methyl donor that is important for a variety of methyltransferase reactions [4]. Moreover, methionine is a precursor in biosynthesis of Hcy and Hcy is produced during the metabolic demethylation of methionine [5]. Since Hcy could not be obtained from diet, metabolic demethylation of methionine is critical for production of Hcy [3]. Studies have shown that Hcy could induce endoplasmic reticulum (ER) stress in human vascular endothelial cells [6], which plays a pivotal role in vascular dysfunction during hypertension [7]. Rennin-angiotensin system (RAS) plays central roles in the pathogenesis of hypertension [1, 8]. In angiotensin (Ang) II-infused rats, hypertension-associated endothelial dysfunction was reduced by alleviation of ER stress [9]. However, the association between hyperhomocysteinemia, renin-angiotensin system (RAS) activation, and ER stress in hypertension remains largely elusive.

In the present study, we established hypertensive rats through high methionine diet feeding and studied the effect of enalapril, a widely used angiotensin converting enzyme inhibitors (ACEi) class antihypertensive drug, on the control of hypertension and ER stress. The results showed that methionine-enriched diet (MED) induced significant hypertension, ER stress, and oxidative stress in rats. The treatment of enalapril could effectively inhibit these pathological changes induced by MED.

## 2. Materials and Methods

### 2.1. Animal Treatment

All the experimental procedures were approved by the Animal Ethics Committee of the Chinese University of Hong Kong. All experiments were carried out in accordance with the approved guidelines. Male Sprague-Dawley rats (180–220 g) were purchased from Laboratory Animal Centre of Fourth Military Medical University. The animals were housed under conditions of controlled temperature (23 ± 2°C) and humidity (60%) with 12 h light/dark cycles. 30 animals were assigned to the following groups: (1) standard diet (SD), (2) methionine-enriched diet (MED), and (3) methionine-enriched diet + enalapril (MED + enalapril). In SD group, rats were fed with standard diet. In the experimental groups, rats were fed from 30th to 90th postnatal day ad libitum or were fed methionine-enriched diet (two-fold standard, 7.7 g/kg). In the MED + enalapril group, rats were treated with enalapril (15 mg/kg/day). Treatment lasted from 60th to 90th postnatal day. Systolic blood pressure (SBP) was recorded every week from 60th to 90th postnatal day. Animals were sacrificed at 91th postnatal day.

### 2.2. Measurement of Systolic Blood Pressure

The systolic blood pressure (SBP) was measured using the tail-cuff blood pressure system (Kent Scientific, Torrington, USA). Arterial blood pressure measurements were carried out at the same time of the day avoiding the influence of the circadian cycle.

### 2.3. Isometric Recording of Aortic Response

Isometric recording of aortic response was conducted as previously described [10]. During equilibration period, the bath fluid was repeatedly changed once in every 15 min. The change in tension was measured by a high-sensitivity isometric force transducer and recorded in a computer using Lab Chart 7 software (PowerLab, AD Instruments, Australia).

### 2.4. Plasma Hcy Determination

Blood was collected, immediately centrifuged, and then stored at −80°C until analysis. The plasma Hcy levels were determined by a commercial kit according to the manufacturer's instructions (Crystal Chem, Inc., IL, USA). The results of Hcy levels were expressed as *μ*mol/L serum.

### 2.5. Plasma ACE Determination

The levels of ACE in plasma were measured using ELISA kits according to the manufacturer's protocols (USCN Life Sciences, Inc., USA). The results of ACE levels were expressed as pg/mL serum.

### 2.6. Western Blot

Aortae were homogenized in ice-cold lysis buffer (50 mM Tris-Cl (pH 7.5), 20 mM NaCl, 5 mM EDTA, 1% TX-100, 0.1% SDS, and 5% glycerol + protease inhibitors) and the lysates were centrifuged at 20,000 g for 20 min at 4°C to collect supernatants. The protein concentration was determined using the BCA assay kit (Thermo Scientific, USA). Protein samples (20 *μ*g) were separated on 1% SDS-10% PAGE and transferred to a polyvinylidene difluoride membrane (Millipore, Billerica, MA, USA). Nonspecific binding sites were blocked by 5% nonfat milk for 1 h at room temperature and then incubated at 4°C overnight with primary antibodies (*β*-actin: Santa Cruz, 1 : 1000; T-PERK: Cell Signaling, 1 : 1000; P-PERK: Cell Signaling, 1 : 1000; P-eIF2*α*: Cell Signaling, 1 : 1000; T-eIF2*α*: Cell Signaling, 1 : 1000; ATF3: Bioworlde, 1 : 500; ATF6: Bioworlde, 1 : 500). After washing 3 times for 20 min each in TBST, the blots were then incubated with appropriate secondary antibodies (Thermo Scientific, 1 : 5000) for 1 h at room temperature and then washed 3 times for 20 min each in TBST. The membranes were finally developed with an enhanced chemiluminescence detection system (ECL reagents, Thermo Scientific, USA). Densitometry was analyzed with Quantity One software (Bio-Rad).

### 2.7. Determination of Oxidative Stress

Aortae were homogenized in cold saline. Content of thiobarbituric acid reaction substances (TBARS) and activities of superoxide dismutase (SOD) and catalase (CAT) in the homogenates were determined using commercial assay kits (Nanjing Jiancheng, China).

### 2.8. Data Analysis

Results represent means ± SEM. Data were analyzed by one-way ANOVA followed by Bonferroni post hoc tests (GraphPad Software, San Diego, USA). *p* < 0.05 indicates statistical difference between groups.

## 3. Results

### 3.1. Enalapril Inhibits MED-Induced Increase of SBP

**Figure 1:**
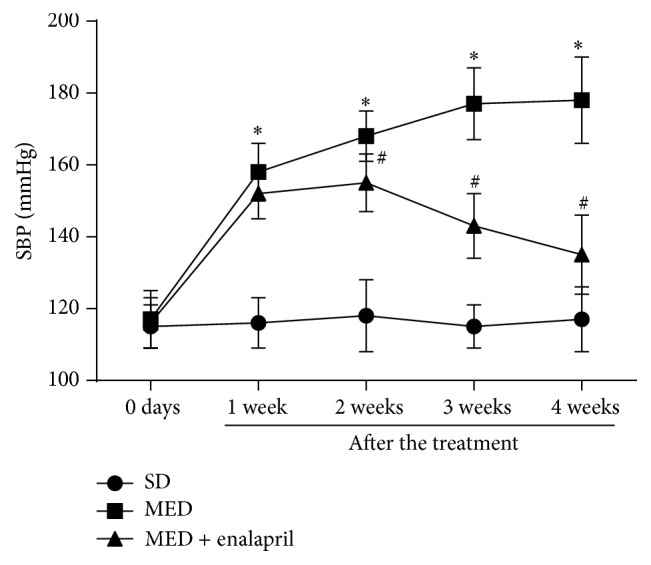
Effect of MED and enalapril on systolic blood pressure in rats. Rats were fed standard diet (SD) or methionine-enriched diet (MED) from 30th to 90th postnatal day with or without enalapril treatment from 60th to 90th postnatal day. Systolic blood pressure (SBP) was recorded every week from 60th to 90th postnatal day. The SBP values at 60–90 postnatal days were shown. ^*∗*^ *p* < 0.05, compared with that of SD rats. ^#^ *p* < 0.05, compared with that of MED rats.

As shown in Figure 1, one-month administration of methionine-enriched diet (MED) significantly increased SBP to 158 ± 11 mmHg in rats. Two-month administration of MED significantly increased SBP to 179 ± 12 mmHg in rats. Compared with that of rats given MED, the administration of enalapril (2–4 weeks) markedly reduced the increase of SBP in rats (Figure 1). The results indicated that MED resulted in hypertension and enalapril could suppress MED-induced hypertension in rats.

### 3.2. Enalapril Inhibits MED-Resulting Increase of Ang II-Induced Contractile Response

**Figure 2:**
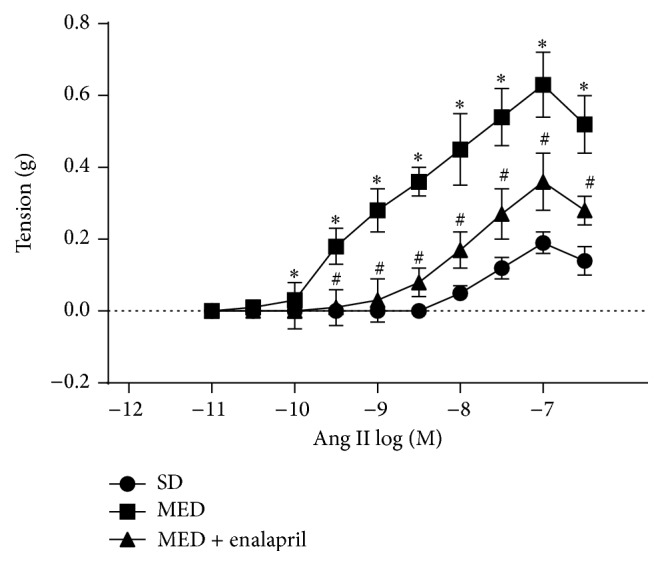
Effect of MED and enalapril on the vascular response to angiotensin II (Ang II) in rats. Rats were fed SD or MED from 30th to 90th postnatal day with or without enalapril treatment from 60th to 90th postnatal day. After the experiment, aorta was collected and Ang II-induced vascular response was determined. ^*∗*^ *p* < 0.05, compared with that of SD rats. ^#^ *p* < 0.05, compared with that of MED rats.

Ang II-induced contractile response in the rat was evaluated. As shown in Figure 2, rings of aorta from rats administrated with SD exhibited concentration-related contractile response to Ang II, with *E*_max_ = 0.19 ± 0.03 g and pD2 values = 8.12 ± 0.13, respectively. In rats fed MED, the efficacy of Ang II was significantly increased, with *E*_max_ = 0.63 ± 0.09 g. In contrast, enalapril administration remarkably reduced the efficacy of Ang II in MED-treated rats, with *E*_max_ = 0.36 ± 0.08 g. These results demonstrated that MED resulted in increase of Ang II-induced contractile response which could be inhibited by enalapril treatment.

### 3.3. Enalapril Inhibits MED-Induced Increase of Plasma Hcy Levels

**Figure 3:**
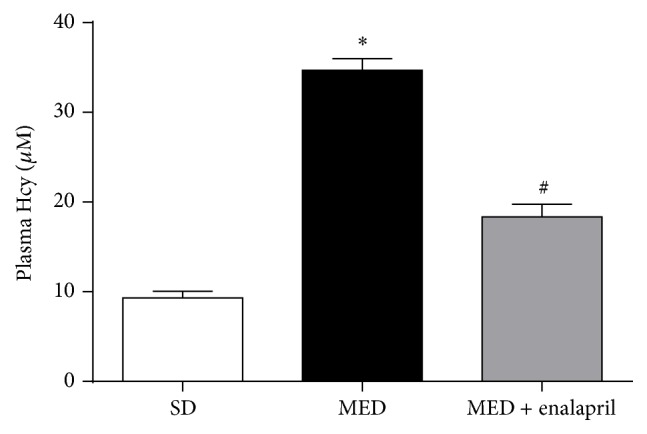
Effect of MED and enalapril on the levels of plasma Hcy in rats. Rats were fed SD or MED from 30th to 90th postnatal day with or without enalapril treatment from 60th to 90th postnatal day. After the experiment, plasma levels of Hcy were measured. ^*∗*^ *p* < 0.05, compared with that of SD rats. ^#^ *p* < 0.05, compared with that of MED rats.

Plasma Hcy levels were examined in the study. In Figure 3, we showed that MED increased the plasma levels of Hcy to more than three times that of SD rats. In addition, the treatment of enalapril significantly inhibited the increase of plasma levels of Hcy induced by MED.

### 3.4. Enalapril Inhibits MED-Induced Increase of Plasma ACE Levels

**Figure 4:**
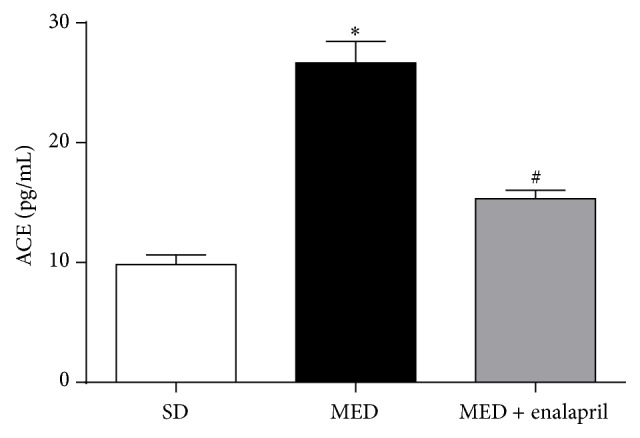
Effect of MED and enalapril on the levels of plasma ACE in rats. Rats were fed SD or MED from 30th to 90th postnatal day with or without enalapril treatment from 60th to 90th postnatal day. After the experiment, plasma levels of ACE were measured. ^*∗*^ *p* < 0.05, compared with that of SD rats. ^#^ *p* < 0.05, compared with that of MED rats.

In the current study, we also evaluated plasma levels of ACE using commercial ELISA kits. As reflected in Figure 4, MED significantly increased plasma levels of ACE to three times that of SD rats. Moreover, the administration of enalapril significantly inhibited the increase of plasma levels of ACE induced by MED.

### 3.5. Enalapril Inhibits MED-Induced ER Stress in Aortae of Rats

**Figure 5:**
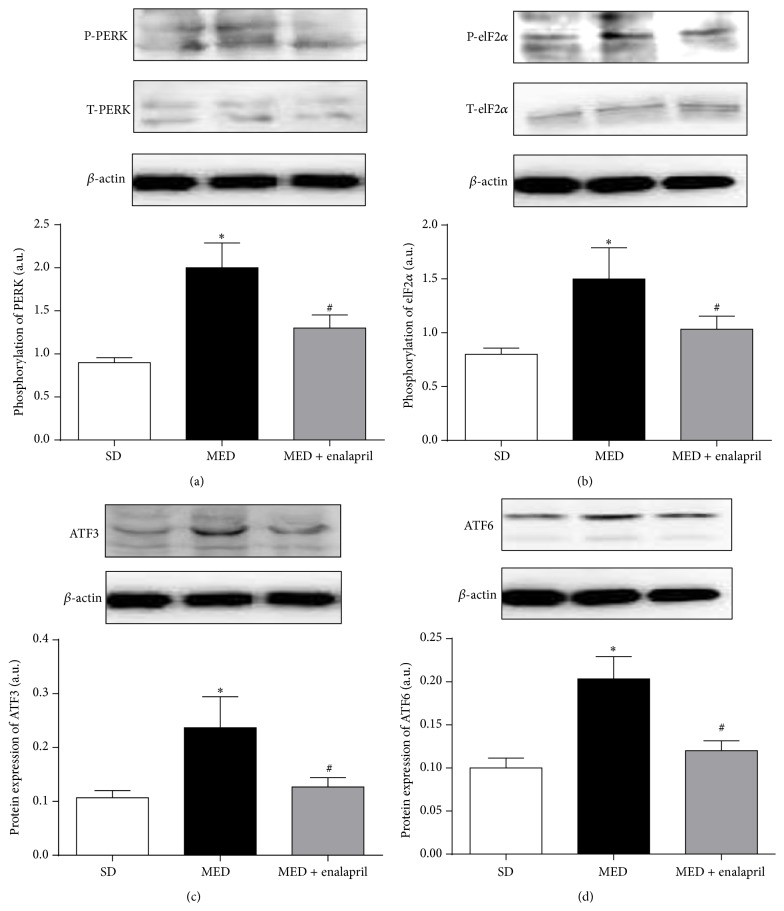
Effect of MED and enalapril on ER stress in aortae of rats. Rats were fed SD or MED from 30th to 90th postnatal day with or without enalapril treatment from 60th to 90th postnatal day. After the experiment, phosphorylation of PERK (a) and eIF2*α* (b) and protein expression of ATF3 (c) and ATF6 (d) were determined by Western blot. Representative blot was shown. Results were also shown as means ± SEM. The bar graphs data resulted from the ratio of density of phosphorylated protein/total protein or target protein/corresponding protein. ^*∗*^ *p* < 0.05, compared with that of SD rats. ^#^ *p* < 0.05, compared with that of MED rats.

In the next step, we assessed the effect of MED and/or enalapril on ER stress in aortae of rats. In Figure 5(a), the results showed that MED significantly increased the phosphorylation of protein kinase R-like endoplasmic reticulum kinase (PERK), which was inhibited by enalapril treatment. Moreover, phosphorylation of eukaryotic initiation factor 2 (eIF2*α*) was enhanced by MED and enalapril treatment significantly suppressed MED-induced increase of phosphorylation of eIF2*α* (Figure 5(b)). Furthermore, MED resulted in significant increase of activating transcription factor (ATF) 3 and ATF6 protein expression in aortae of rats (Figures 5(c) and 5(d)). Enalapril treatment markedly inhibited the increase of ATF3 and ATF6 protein expression resulting from MED (Figures 5(c) and 5(d)). The results indicated that MED resulted in ER stress in aortae of rats which could be ameliorated by enalapril.

### 3.6. Enalapril Inhibits MED-Induced Oxidative Stress in Rats

**Figure 6:**
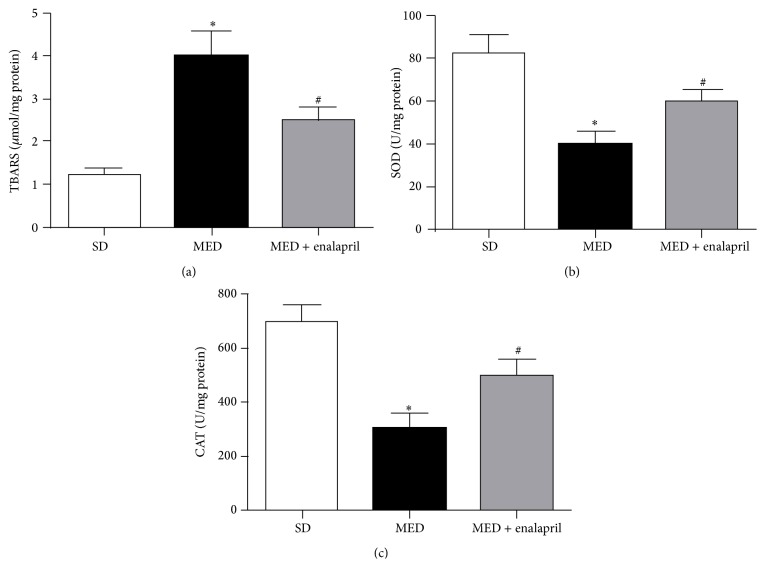
Effect of MED and enalapril on oxidative stress in rats. Rats were fed SD or MED from 30th to 90th postnatal day with or without enalapril treatment from 60th to 90th postnatal day. After the experiment, plasma content of TBARS (a) and activities of SOD (b) and CAT (c) were measured by commercial kits according to the protocols. ^*∗*^ *p* < 0.05, compared with that of SD rats. ^#^ *p* < 0.05, compared with that of MED rats.

Next, we evaluated oxidative stress in rats. As illustrated in Figure 6(a), serum TBARS content was significantly increased by MED. Enalapril markedly inhibited MED-induced increase of TBARS level. Moreover, SOD and CAT activities were determined. The results showed that MED resulted in a significant decrease of SOD and CAT activities in rats (Figures 6(b) and 6(c)). The treatment of enalapril markedly increased the activities of SOD and CAT, compared with that in MED group (Figures 6(b) and 6(c)). The results demonstrated that MED resulted in significant oxidative stress which could be inhibited by enalapril treatment.

## 4. Discussion

Increased blood Hcy level is considered to be an independent risk factor for the pathogenesis of hypertension [3]. However, the causal relationship between the increase of Hcy and hypertension is limited and the possible mechanism needs to be clarified. In the present study, we investigated the effect of MED on blood pressure in rats. The results showed that MED induced significant increase of SBP and Ang II-induced contractile response in aortae of rats. These data demonstrated that MED resulted in hypertension in rats.

It is noted that rennin-angiotensin system (RAS) plays a central role in the pathogenesis of hypertension [1, 8]. To examine the possible role of angiotensin system in high Hcy-induced hypertension, MED rats were treated by enalapril, an inhibitor of ACE. The results showed that enalapril significantly inhibited MED-induced increase of SBP and Ang II-induced contractile response in aortae of rats. Moreover, MED resulted in significant increase of ACE content in rats. Consistently, previous studies have shown that ACE inhibitors could decrease Hcy levels in essential hypertension [11]. These data suggested that activation of ACE may be involved in the pathogenesis of high Hcy-associated hypertension.

The ER is a multifunctional intracellular organelle that plays important roles in various physiological and pathophysiological processes [12]. In response to numerous cellular stresses, the function of ER could easily be influenced, resulting in the misfolding and aggregation of proteins within the lumen of the ER, causing ER stress [13]. Once ER stress occurs, a complex cellular signaling network known as the unfolded protein response (UPR) could be initiated, leading to the activation of reparative signaling, including PERK, eIF2*α*, ATF3, and ATF6 [14, 15]. Chronic ER stress has been considered to be a major contributor to pathophysiological changes in cardiovascular diseases [16]. In contrast, pharmacological inhibition of ER stress has been shown to improve various cardiovascular abnormalities [17, 18]. Moreover, suppression of ER stress has been shown to improve endothelium-dependent contraction in aortae of spontaneously hypertensive rats [19]. Consistent with these results, in the present study, we also found that MED-induced hypertension was accompanied by the occurrence of ER stress, as evidenced by the increase of phosphorylation of PERK and eIF2*α* and increased protein expression level of ATF3 and ATF6. In addition, the treatment of enalapril significantly inhibited MED-resulting ER stress in aortae of rats. Previous investigations have shown that Ang II is able to induce ER stress [20] and ER stress mediates the effects of angiotensin in vivo and in vitro [20, 21]. ER stress was also reported to mediate Hcy-induced endothelial dysfunction [22, 23]. In combination with previous results, the data demonstrated that activation of ACE was involved in MED-induced ER stress in aortae of rats.

Under various pathological conditions, ER stress and oxidative stress mutually interacted, leading to severe adverse outcomes [24, 25]. It is suggested that oxidative stress plays important roles in ER stress-mediated vascular endothelial dysfunction [26, 27]. In the current study, we also found that MED resulted in significant oxidative stress, as reflected by increase of TBARS and decrease of SOD and CAT activities. Previous studies have shown that enalapril could improve oxidative stress in kidney of spontaneously hypertensive rats [28]. Consistently, we showed that MED-resulting oxidative stress could be ameliorated by enalapril. Furthermore, we discovered that enalapril could reduce the increase of Hcy levels in rats induced by MED. These results demonstrated that MED may exert a positive loop between the activation of ACE and accumulation of Hcy, aggravating the high Hcy-associated hypertension.

**Figure 7:**
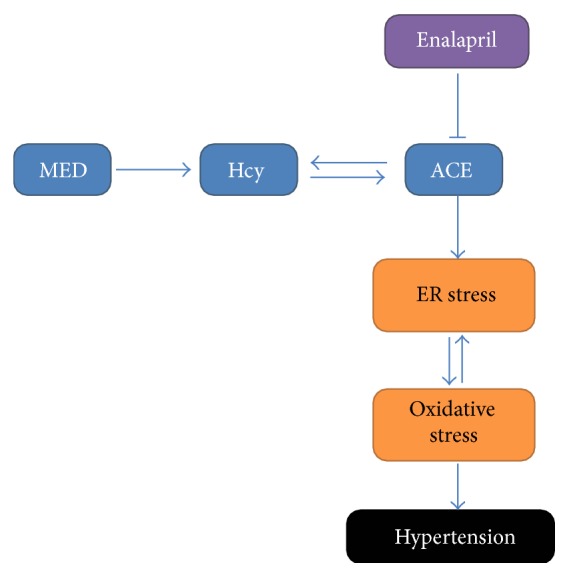
Mechanism of the protective effect of enalapril against MED-induced hypertension.

In conclusion, the present study examined the causal relationship between high level of Hcy and hypertension and explored the possible mechanisms. We found that MED increased Hcy level, activated ACE, and then induced ER stress and oxidative stress, leading to the increase of SBP (Figure 7). The treatment of enalapril could ameliorate these changes effectively. The data provide novel insights into the mechanism of high Hcy-associated hypertension and the therapeutic efficiency of enalapril.

## References

[1] Chobanian A. V., Bakris G. L., Black H. R. (2003). The seventh report of the joint national committee on prevention, detection, evaluation, and treatment of high blood pressure: the JNC 7 report. *The Journal of the American Medical Association*.

[2] Refsum H., Ueland P. M., Nygård O., Vollset S. E. (1998). Homocysteine and cardiovascular disease. *Annual Review of Medicine*.

[3] Sutton-Tyrrell K., Bostom A., Selhub J., Zeigler-Johnson C. (1997). High homocysteine levels are independently related to isolated systolic hypertension in older adults. *Circulation*.

[4] Selhub J. (1999). Homocysteine metabolism. *Annual Review of Nutrition*.

[5] Hoffer L. J. (2004). Homocysteine remethylation and trans-sulfuration. *Metabolism*.

[6] Outinen P. A., Sood S. K., Pfeifer S. I. (1999). Homocysteine-induced endoplasmic reticulum stress and growth arrest leads to specific changes in gene expression in human vascular endothelial cells. *Blood*.

[7] Hasty A. H., Harrison D. G. (2012). Endoplasmic reticulum stress and hypertension—a new paradigm?. *Journal of Clinical Investigation*.

[8] He W. J., Li C., Rao D. C. (2015). Associations of renin-angiotensin-aldosterone system genes with blood pressure changes and hypertension incidence. *American Journal of Hypertension*.

[9] San C. W., Yuen N. C., Yen T. Y. (2015). Black tea protects against hypertension-associated endothelial dysfunction through alleviation of endoplasmic reticulum stress. *Scientific Reports*.

[10] Waghe P., Sarath T. S., Gupta P. (2015). Arsenic causes aortic dysfunction and systemic hypertension in rats: augmentation of angiotensin II signaling. *Chemico-Biological Interactions*.

[11] Poduri A., Kaur J., Thakur J. S., Kumari S., Jain S., Khullar M. (2008). Effect of ACE inhibitors and beta-blockers on homocysteine levels in essential hypertension. *Journal of Human Hypertension*.

[12] Lavoie C., Roy L., Lanoix J. (2011). Taking organelles apart, putting them back together and creating new ones: lessons from the endoplasmic reticulum. *Progress in Histochemistry and Cytochemistry*.

[13] Lai E., Teodoro T., Volchuk A. (2007). Endoplasmic reticulum stress: signaling the unfolded protein response. *Physiology*.

[14] Ashraf N. U., Sheikh T. A. (2015). Endoplasmic reticulum stress and oxidative stress in the pathogenesis of non-alcoholic fatty liver disease. *Free Radical Research*.

[15] Scheper W., Hoozemans J. J. (2015). The unfolded protein response in neurodegenerative diseases: a neuropathological perspective. *Acta Neuropathologica*.

[16] Gotoh T., Endo M., Oike Y. (2011). Endoplasmic reticulum stress-related inflammation and cardiovascular diseases. *International Journal of Inflammation*.

[17] Groenendyk J., Sreenivasaiah P. K., Kim D. H., Agellon L. B., Michalak M. (2010). Biology of endoplasmic reticulum stress in the heart. *Circulation Research*.

[18] Glembotski C. C. (2008). The role of the unfolded protein response in the heart. *Journal of Molecular and Cellular Cardiology*.

[19] Spitler K. M., Matsumoto T., Webb R. C. (2013). Suppression of endoplasmic reticulum stress improves endothelium-dependent contractile responses in aorta of the spontaneously hypertensive rat. *The American Journal of Physiology—Heart and Circulatory Physiology*.

[20] Wang J., Wen Y., Lv L. (2015). Involvement of endoplasmic reticulum stress in angiotensin II-induced NLRP3 inflammasome activation in human renal proximal tubular cells in vitro. *Acta Pharmacologica Sinica*.

[21] Young C. N., Li A., Dong F. N., Horwath J. A., Clark C. G., Davisson R. L. (2015). Endoplasmic reticulum and oxidant stress mediate nuclear factor-*κ*B activation in the subfornical organ during angiotensin II hypertension. *American Journal of Physiology—Cell Physiology*.

[22] Wu S., Gao X., Yang S., Meng M., Yang X., Ge B. (2015). The role of endoplasmic reticulum stress in endothelial dysfunction induced by homocysteine thiolactone. *Fundamental & Clinical Pharmacology*.

[23] Wang X. C., Sun W. T., Yu C. M. (2015). ER stress mediates homocysteine-induced endothelial dysfunction: modulation of IKCa and SKCa channels. *Atherosclerosis*.

[24] Volgyi K., Juhász G., Kovacs Z., Penke B. (2015). Dysfunction of endoplasmic reticulum (ER) and mitochondria (MT) in Alzheimer's disease: the role of the ER-MT cross-talk. *Current Alzheimer Research*.

[25] Kim B., Kim H. S., Jung E.-J. (2015). Curcumin induces ER stress-mediated apoptosis through selective generation of reactive oxygen species in cervical cancer cells. *Molecular Carcinogenesis*.

[26] Galán M., Kassan M., Kadowitz P. J., Trebak M., Belmadani S., Matrougui K. (2014). Mechanism of endoplasmic reticulum stress-induced vascular endothelial dysfunction. *Biochimica et Biophysica Acta: Molecular Cell Research*.

[27] Lenna S., Han R., Trojanowska M. (2014). Endoplasmic reticulum stress and endothelial dysfunction. *IUBMB Life*.

[28] Chandran G., Sirajudeen K. N., Nik Yusoff N. S., Swamy M., Samarendra M. S. (2014). Effect of the antihypertensive drug enalapril on oxidative stress markers and antioxidant enzymes in kidney of spontaneously hypertensive rat. *Oxidative Medicine and Cellular Longevity*.

